# Molecular insights into the interaction of hemorphin and its targets

**DOI:** 10.1038/s41598-019-50619-w

**Published:** 2019-10-14

**Authors:** Amanat Ali, Bincy Baby, Soja Saghar Soman, Ranjit Vijayan

**Affiliations:** 1 Department of Biology, College of Science, United Arab Emirates University, PO Box 15551, Al Ain, Abu Dhabi United Arab Emirates; 2 New York University Abu Dhabi, PO Box 129188, Abu Dhabi, United Arab Emirates

**Keywords:** Protein function predictions, Molecular modelling

## Abstract

Hemorphins are atypical endogenous opioid peptides produced by the cleavage of hemoglobin beta chain. Several studies have reported the therapeutic potential of hemorphin in memory enhancement, blood regulation, and analgesia. However, the mode of interaction of hemorphin with its target remains largely elusive. The decapeptide LVV-hemorphin-7 is the most stable form of hemorphin. It binds with high affinity to mu-opioid receptors (MOR), angiotensin-converting enzyme (ACE) and insulin-regulated aminopeptidase (IRAP). In this study, computational methods were used extensively to elucidate the most likely binding pose of mammalian LVV-hemorphin-7 with the aforementioned proteins and to calculate the binding affinity. Additionally, alignment of mammalian hemorphin sequences showed that the hemorphin sequence of the camel harbors a variation – a Q > R substitution at position 8. This study also investigated the binding affinity and the interaction mechanism of camel LVV-hemorphin-7 with these proteins. To gain a better understanding of the dynamics of the molecular interactions between the selected targets and hemorphin peptides, 100 ns molecular dynamics simulations of the best-ranked poses were performed. Simulations highlighted major interactions between the peptides and key residues in the binding site of the proteins. Interestingly, camel hemorphin had a higher binding affinity and showed more interactions with all three proteins when compared to the canonical mammalian LVV-hemorphin-7. Thus, camel LVV-hemorphin-7 could be explored as a potent therapeutic agent for memory loss, hypertension, and analgesia.

## Introduction

Initial efforts in the 1980s to recognize endogenously produced opioid peptides led to the characterization of hemoglobin-derived (Hb-derived) peptides that have opiate-like activity^1^. These peptides are short, 4–10 amino acids in length and are released during physiological or physiopathological hemoglobin beta-chain proteolytic degradation^2^. These Hb beta chain derived peptides, named hemorphins, share a central tetrapeptide core (Tyr-Pro-Trp-Thr) from the N-terminal region. Interestingly, both N- and C-terminal extensions of hemorphin have been isolated from human and bovine tissue^3^. Hemorphin peptides of varying lengths can be produced by the degradation of hemoglobin beta chain through numerous enzymatic digestions including lysosomal proteases, pepsin, chymotrypsin-like proteases, aspartic endoprotease cathepsin and macrophagic enzymes^4–6^. It has been reported that the inhibition of intraerythrocytic proteasome blocked the generation of hemorphin, supporting the involvement of proteasomes in the processing and generation of hemorphin^7^.

The first characterized hemoglobin derived opioid peptide was hemorphin-4 (YPWT). Hemorphin-4 was initially isolated from bovine blood treated with a mixture of gastrointestinal enzymes^1^. Later, a series of peptides, including hemorphin-4 to -7 and LVV-hemorphin-4, -6, and -7, were identified that contained the YPWT sequence^8–10^. These are naturally occurring peptides produced in the brain, spinal cord, plasma and cerebrospinal fluid^5,11–14^. Hemorphins have been shown to possess numerous biological activities including effects on spatial learning, inflammation, analgesia and transient hypotension^15–18^. Among hemorphin peptides, LVV-hemorphin-7 (LVV-H7 or LVVYPWTQRF) is the longest with 10 amino acids. Functional activity of LVV-hemorphin-7 derived from either Hb β, γ, δ and ε chains have been widely studied. Several reports have indicated that LVV-hemorphin-7 is the most abundant and has the highest hydrophobicity in mammalian central nervous system (CNS)^13,19^.

The discovery of stereospecific opioid binding sites in the mammalian brain initiated a series of studies to identify their natural ligands. Met- and Leu-enkephalin were the first described endogenous opioid peptides^20^. Later, various studies revealed the existence of new classes of bioactive peptides produced from limited proteolysis of protein precursors. Enkephalins, dynorphins, and beta-endorphin are the classical opioid peptides released by the cleavage of proenkephalin, prodynorphin and proopiomelanocortin, respectively, and are expressed in the CNS^14,21,22^. Mu, delta and kappa are three opioid receptors that have been investigated extensively. Partial proteolysis of proteins leads to the production of several groups of peptides. For instance, cytochrophins are released from mitochondrial cytochrome b, casomorphins from the milk protein beta-casein, and hemorphins from hemoglobin^1,3^. Agonistic binding to opioid receptors produces profound analgesia in inflamed tissue, highlighting the importance of investigating such molecules^23^. It has been shown that all hemorphin peptides, including LVV-hemorphin-7, show an affinity for opioid receptors^5,12^. Additionally, hemorphin-5, -6, and -7 have shown opioid-like activity in electrically stimulated myenteric plexus/longitudinal muscle preparation of the guinea pig ileum. Glucose phosphate isomerase (GPI) bioassay had also shown agonistic opioid-like effects of different hemorphins^24^. Further, hemorphin’s opiod receptor affinity was investigated in rat brain membrane and results showed IC_50_ values in the micromolar range^24^. Moreover, it has also been suggested that hemorphins imitate opioid peptides in terms of their analgesic activities. Hemorphins have also produced a dose-dependent antinociceptive response in mice^25^. Chow and colleagues reported that intrathecal doses of angiotensin IV (Ang IV) and LVV-hemorphin-7 cause anti-hyperalgesia in rats^26^.

The renin-angiotensin system (RAS) is one of the most thoroughly investigated enzyme-neuropeptide systems. RAS originated from the classical renin/angiotensin-converting enzyme (ACE)/angiotensin II (Ang II)/angiotensin II type I (AT1) receptor axis and has a physiological role in the regulation of cardiovascular and renal function, aldosterone biosynthesis and release, blood pressure and body salt, and fluid balance^27^. In recent years, a lot of progress has been made in identifying new proteins of this system as well as elucidating their roles and signal transduction mechanisms. Therefore, renin/ACE/Ang II/AT1 and AT2 axis are not the exclusive signaling pathways for the system. The prorenin/PRR/MAP kinases ERK1/2 axis, the ACE2/Ang (1–7)/Mas receptor axis and the Ang IV/AT4/IRAP (insulin-regulated aminopeptidase, IRAP) axis are three new axes that have been reported recently^28,29^. LVV-hemorphin-7’s role in blood pressure regulation has already been suggested by several studies. Intraperitoneal injection of LVV-hemorphin-7 was found to cause a substantial decrease in blood pressure and heart rate in hypertensive rats^30^. LVV-hemorphin-7 also potentiated the hypotensive effect of bradykinin in rats^31^. LVV-hemorphin-7 also inhibited ACE, a key component of RAS, indicating a major role in the regulation of blood pressure^32^.

RAS in the brain is believed to be involved in processes other than homeostatic control. It has been associated with neuronal differentiation, nerve regeneration, learning and memory^33^. Various physiological functions have been associated with Ang IV, including the facilitation of memory^34^. LVV-hemorphin-7 was identified as an endogenous high-affinity ligand of the presumed angiotensin IV receptor (AT4R)^19^. Further characterization of this putative receptor suggested that the protein was analogous to IRAP, a type II integral membrane protein whose catalytic activity is inhibited by LVV-hemorphin-7 and Ang IV^35–37^. *In vivo* and *in vitro* studies revealed that LVV-hemorphin-7 could play a potential role in learning and memory. An intracerebral administration of LVV-hemorphin-7 enhanced spatial learning in rats and diminished the effects of scopolamine-induced learning deficits in spatial learning tests and fear conditioning^17,38^. LVV-hemorphin-7 has also been shown to modify the behavior of rats. Additionally, reduced depression and increased locomotion, induced by LVV-hemorphin-7, depended on the activation of oxytocin receptors most likely due to changes in the oxytocinase activity of AT4R^39^. It was assumed that by inhibiting IRAP activity, LVV-hemorphin-7 protects the substrates of IRAP such as oxytocin and vasopressin, which are known to play a key role in memory and learning^40^. *In vivo* microdialysis revealed that LVV-hemorphin-7 enhanced spatial working memory without a significant increase in blood flow or hippocampal glucose uptake^41^.

*In vivo* and *in vitro* studies have demonstrated the potential therapeutic role of hemorphin in several pathological conditions. However, the precise binding behavior of hemorphin on target proteins have not been clearly elucidated yet. In this study, using *in silico* techniques, we provide early insight into the binding of LVV-hemorphin-7 with MOR, ACE and IRAP. Determination of the likely binding mode of peptide is a difficult task and protein-peptide docking is often used for identifying potential modes of interactions. Molecular docking generates several possible binding solutions, and the most appropriate binding pose is frequently judged by comparison of docking scores^42,43^. With the advent of high performance and rigorous methods, binding free energy calculations and long timescale MD simulations could be employed to study protein-peptide interactions at an atomic level^44–47^. In this study, protein-peptide docking and binding free energy calculations using molecular mechanics-generalized Born surface area (MM-GBSA) were used to identify the most probable binding mode of LVV-hemorphin-7 in these proteins. Additionally, the solvation effects were examined by using explicit solvent MD simulations. The hemorphin sequence is highly conserved among mammals. However, multiple sequence alignment of hemoglobin beta protein from several mammals showed that the camel hemorphin sequence harbors one variation. This study explored both variants of LVV-hemorphin-7 - camel and other mammals (referred to as non-camel here onwards) - to identify the differences in binding mode, stability and interactions.

## Results

### Multiple sequence alignment of hemoglobin beta protein sequences

**Figure 1:**
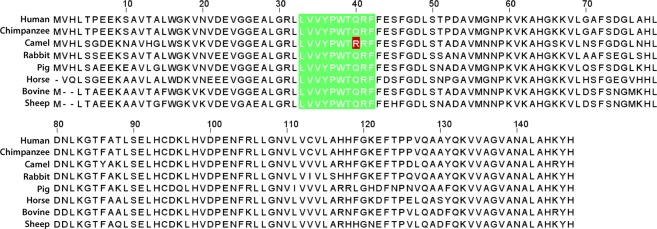
Multiple sequence alignment of hemoglobin beta protein sequences from closely related mammals - *Homo sapiens* (human), *Pan troglodytes* (chimpanzee), *Camelus dromedarius* (camel), *Oryctolagus cuniculus* (rabbit), *Sus scrofa* (wild pig), *Equus caballus* (horse), *Bos taurus* (bovine), and *Ovis aries* (sheep).

Hemoglobin beta (HBB) protein reference sequences of closely related mammals were retrieved from NCBI protein database. Multiple sequence alignment was performed using ClustalW to identify the similarities and differences in the HBB protein sequences. The decapeptide LVV-hemorphin-7 sequence is highly conserved among mammals. In human, chimpanzee, camel, pig, and rabbit it lies between amino acid positions 32 to 41 in the HBB protein sequence. In the bovine and sheep protein, it ranges from amino acids 30 to 39 and in the horse the decapeptide is located between amino acids 31 to 40. The sequence comparison clearly showed a single amino acid substitution in the camel HBB protein at position 40, where a glutamine has been changed to an arginine (Q > R). All other residues are identical (Fig. 1).

### Structure-based homology modeling of mu-type opioid receptor

**Figure 2:**
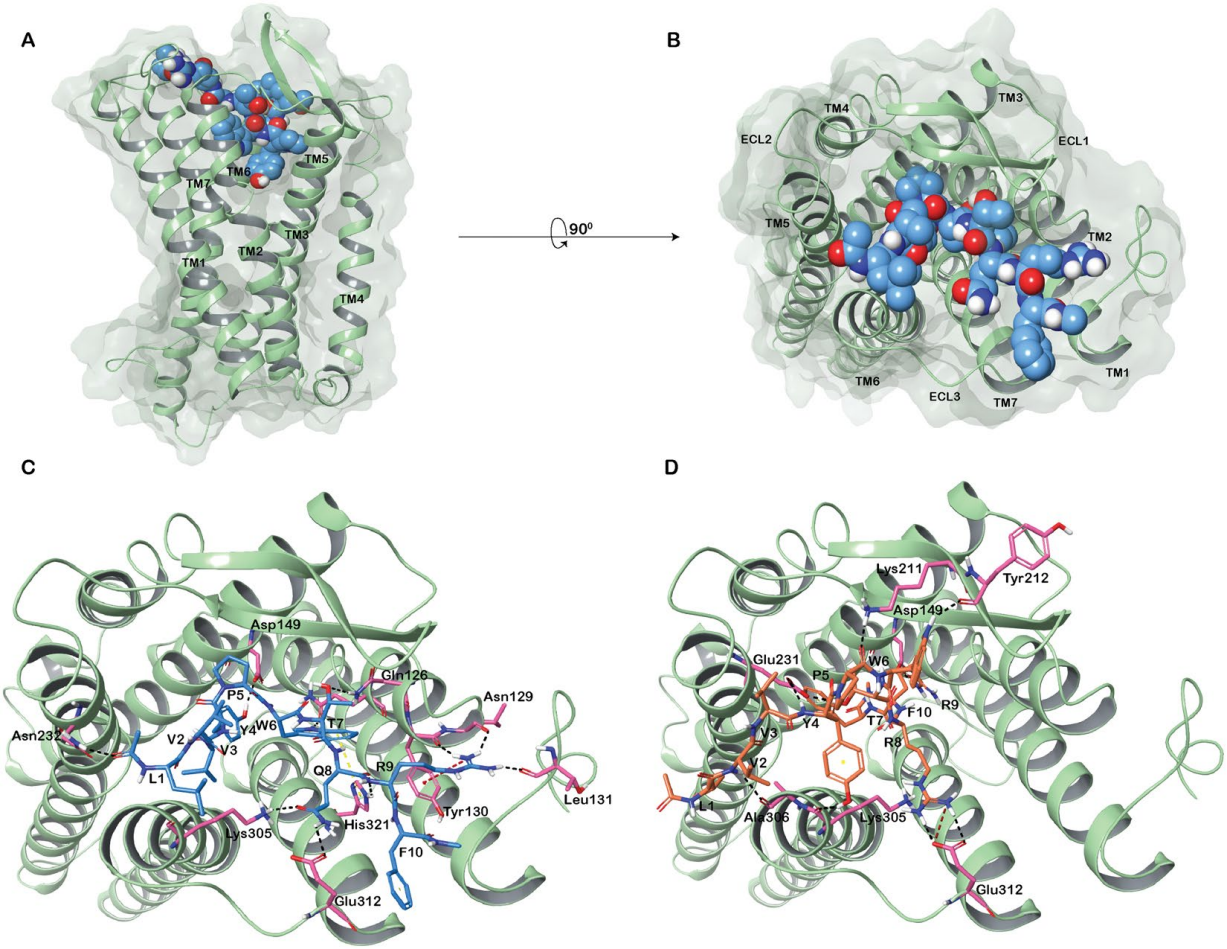
Modeled active human MOR. (**A**) Side view (**B**) Top view. (**C**) LVVYPWTQRF (non-camel LVV-hemorphin-7) docked in the binding pocket of the putative active conformation of human MOR. (**D**) LVVYPWTRRF (camel LVV-hemorphin-7) docked in the binding pocket of the putative active conformation of human MOR. Hydrogen bonds are represented by black dotted lines and π-π stacking by yellow dotted lines.

The three-dimensional homology model of the active state of the human mu-type opioid receptor (MOR) (UniProt accession: P35372) was generated using the X-ray crystallographic structure of MOR from *Mus musculus* (PDB ID: 5C1M) (Fig. 2A). Structural model of the active conformation of MOR is analogous to the conserved seven-transmembrane topology of G protein-coupled receptors (GPCRs). The Ramachandran plot showed that the overall stereochemical quality of the generated model was good with almost all the residues in the favorable and allowed regions (Supplementary Fig. 1).

### Molecular docking

By means of exhaustive docking and free energy calculations, the binding mode of both non-camel (LVVYPWTQRF) and camel (LVVYPWTRRF) LVV-hemorphin-7 peptides were identified. The binding free energies were obtained for each binding mode with the molecular mechanics generalized Born and surface area (MM-GBSA) method.

#### Mu-opioid receptor

The docking of both camel and non-camel LVV-hemorphin-7 with MOR generated 10 models each. The best-docked pose of non-camel LVV-hemorphin-7, which was in the active site of MOR, produced a Glide docking score, GlideScore (GScore), of −10.88 kcal/mol and an MM-GBSA binding energy of −97.82 kcal/mol (Table 1). The best-docked conformation of non-camel LVV-hemorphin-7 revealed that the N-terminal residues of the peptide interacted with residues in transmembrane helices 5 and 6 (TM5 and TM6) lining the bottom of the binding site. The first three residues (Leu1-Val2-Val3) of the peptide produced extensive hydrophobic interactions with TM6 residues Ile298, Tyr301, Val302, Ala306, and Val308. The N-terminal leucine formed a hydrogen bond with the backbone amino group of the hydrophilic Asn232 present on TM5. Tyr4 in the non-camel LVV-hemorphin-7 interacted with Asp149 through a hydrogen bond and, along with Pro5, it produced hydrophobic interactions with Ile146 and Tyr150 present on TM3. Pro5, Trp6, and Thr7 interacted with Arg213, Ile217, Cys219, Thr220, and Leu221 on the beta sheet. Hydrophobic interactions were formed between the C-terminal residues (Thr7-Gln8-Arg9-Phe10) and Trp320, His321, Ile324, and Tyr328 on TM7. Trp6 and Gln8 interacted via π- π stacking and hydrogen bond, respectively, with His321 on TM7. Gln8 formed additional hydrogen bonds with the side chain of the Glu312 present on the third extracellular loop and with Lys305 on TM5 (Fig. 2C and Supplementary Fig. 2A).

**Table 1:** Interacting residues of the best pose of non-camel (LVVYPWTQRF) and camel (LVVYPWTRRF) LVV-hemorphin-7 with MOR, ACE, and IRAP.

Protein	Peptide	Glide docking score – GScore (kcal/mol)	MM-GBSA (kcal/mol)	Residues forming hydrogen bonds	Residues forming hydrophobic interactions	Residues forming π interactions
MOR	LVVYPWTQRF	−10.88	−97.82	Gln126, Asn129, Leu131, Asp149, Asn232, Lys305, Glu312, His321	Leu58, Pro61, Pro65, Tyr77, Tyr130, Met132, Ile146, Tyr150, Ile217, Cys219, Leu221, Trp228, Ile298, Tyr301, Val302, Ala306, Val308, Trp320, Ile324, Tyr328	His321
MOR	LVVYPWTRRF	−10.53	−115.08	Asp149, Lys211, Tyr212, Glu231, Lys305, Ala306, Glu312	Ile146, Tyr150, Cys219, Leu221, Phe223, Trp228, Leu234, Ile236, Val238, Ile298, Val302, Ile303, Leu307, Val308, Trp320, Ile324, Tyr328	
ACE	LVVYPWTQRF	−10.66	−119.32	His331, Ala332, Ala334, Gln355, Glu362, Tyr501	Val36, Tyr111, Leu139, Phe178, Tyr186, Tyr197, Trp198, Trp201, Trp257, Val329, Cys330, Ala335, Tyr338, Tyr369, Tyr372, Pro385, Phe435, Pro497, Val495, Tyr501, Phe505	His388, Phe490
ACE	LVVYPWTRRF	−12.31	−151.57	Asp140, Tyr186, Tyr197, Ala332, Tyr369, Glu389	Leu32, Val36, Ala94, Leu98, Ala101, Tyr111, Leu115, Phe178, Tyr197, Trp201, Cys330, Ala332, Cys348, Ala334, Trp335, Phe435, Phe490, Val495, Tyr498, Tyr501, Phe505	His388
IRAP	LVVYPWTQRF	−14.86	−148.04	Gly428, Glu441, Lys460, Glu509, Asp510, Glu541, Glu818, Glu825, Glu895	Tyr272, Pro296, Ala427, Ala429, Met430, Ala453, Leu457, Ile461, Tyr495, Ala514, Phe544, Tyr549, Phe550, Ala763, Leu769, Ala822, Phe826, Tyr961	
IRAP	LVVYPWTRRF	−13.65	−163.03	Gly428, Glu441, Asp510, Glu541, Glu818, Glu825, Glu895	Tyr272, Pro296, Ala427, Ala429, Met430, Ala453, Leu457, Ile461, Leu469, Tyr495, Ala514, Phe544, Tyr549, Phe550, Ala822, Phe826, Pro957, Tyr961	

The best pose of camel LVVYPWTRRF had a GScore of −10.53 kcal/mol and MM-GBSA binding energy of −115.08 kcal/mol (Table 1). The N-terminal residues of camel LVV-hemorphin-7 interacted with residues present on TM5 (Glu231 and Ile236) and TM6 (Val302, Ile303, and Leu307). Differing from non-camel LVV-hemorphin-7, the residues at positions 5 and 6 (Pro5 and Trp6) formed hydrogen bonds with Tyr212 and Lys211, respectively, and produced hydrophobic interaction with the β-sheet residues. The C-terminal residues interacted with residues on TM3, TM5 and TM7. Arg9 also formed a hydrogen bond with Asp149 on TM3 and the substituted arginine at position 8 produced a hydrogen bond with the side chain of Glu312 on the third extracellular loop. Additionally, Glu231 on TM5 produced hydrogen bonds with Val3, Tyr4 and Thr7 (Fig. 2D). Hydrophobic interactions are shown in Supplementary Fig. 2B.

#### Angiotensin-converting enzyme

The binding mode and the affinity of the non-camel and the camel LVV-hemorphin-7 with ACE were predicted using molecular docking and binding free energy calculations. Table 1 lists the binding scores and the ACE amino acids that interacted with the peptides. The positioning of both non-camel and camel LVV-hemorphin-7 in the ACE active site was well defined, encompassing the S1 and S2 subsites. It is evident from the 2D ligand interaction diagrams that an extensive network of hydrogen bonds, hydrophobic, and electrostatic interactions were formed between ACE and hemorphin peptides (Supplementary Fig. 3).

**Figure 3:**
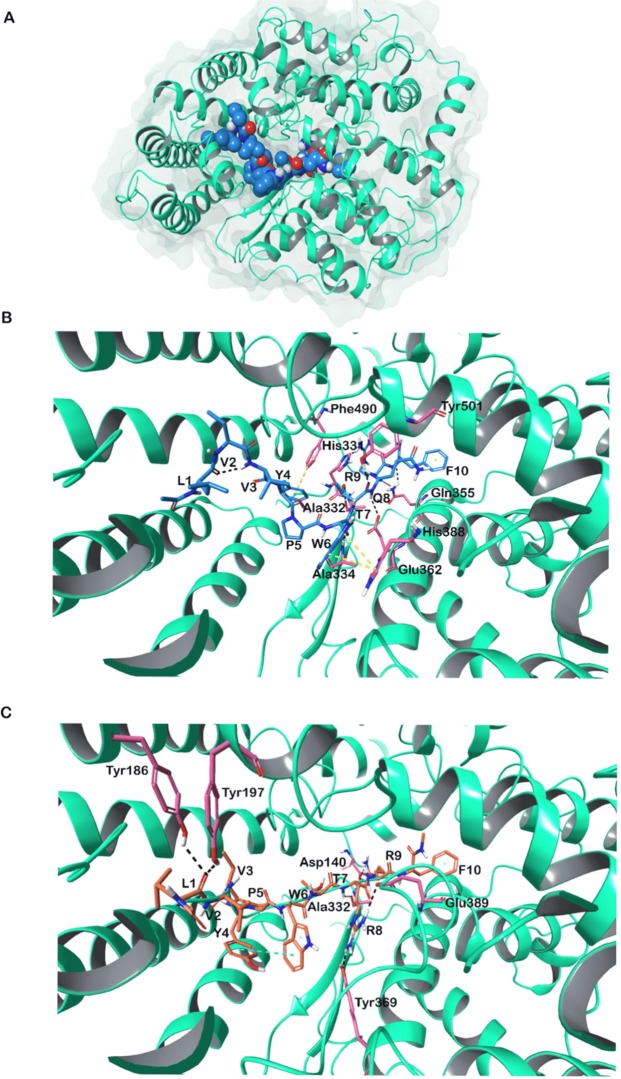
(**A**) Three dimensional structure of ACE. (**B**) LVVYPWTQRF (non-camel LVV-hemorphin-7) docked in the active site of ACE. (**C**) LVVYPWTRRF (camel LVV-hemorphin-7) docked in the active site of ACE. Hydrogen bonds are represented by black dotted lines and π-π stacking represented by yellow dotted lines.

The best binding pose of non-camel LVVYPWTQRF had a GScore of −10.66 kcal/mol. The MM-GBSA binding energy calculated for this pose was −119.32 kcal/mol. The N-terminal of LVV-hemorphin-7 extended towards the lid of the active site. The C-terminal bound deep in the active site of ACE with Arg9 binding in the S2 subsite with its side chain oriented towards S1′. The sidechain of Arg9 formed a hydrogen bond with His331 in the S2 subsite and electrostatic interaction with Asp140 in S1′. Thr7 and Gln8 occupied the S1 subsite and Gln8 made hydrophobic interactions with Phe435 and Phe505. The backbone amino and carboxyl group of Thr7 formed hydrogen bonds with Glu362, and Tyr501 respectively and the side chain formed a hydrogen bond with Ala332 in the S1 subsite. The backbone carboxyl group of Trp6 formed a hydrogen bond with Ala334 and the side chain formed a π-π interaction with His388. It also produced hydrophobic interactions with Trp335, Tyr338, Tyr369, Tyr372, and Pro385. Residues at 4 and 5 (Tyr4 and Pro5) showed hydrophobic interactions with Val329, Pro385, Val495 and Pro497 and a π-π stacking was formed between Tyr4 and Phe490. The N-terminal residues oriented towards the lid and showed hydrophobic interactions with Tyr111, Phe178, Tyr186, Tyr197, Trp198, and Trp201 (Fig. 3B and Supplementary Fig. 3A).

Binding mode of LVVYPWTRRF in the ACE active site was different from the non-camel peptide. More specifically, the positioning of the C-terminal in S1′ and S2 pockets was different. The best docked pose had a GScore of −12.31 kcal/mol and an MM-GBSA binding energy of −151.57 kcal/mol (Table 1). Arg9 and Phe10 were stabilized in the S2 subsite, through hydrophobic interactions with Phe435, Tyr498, Tyr501, and Phe505. The side chain of Arg9 interacted with the S1′ subsite and formed a hydrogen bond with Asp140. Interestingly, the arginine at position 8 in the camel LVV-hemorphin-7 bound in the S1 subsite and produced more interactions with the active site residues. The side chain of Arg8 made hydrogen bonds with Tyr369 and Glu389 and showed hydrophobic interaction with Ala334. The backbone amino group of Thr7 interacted with Ala332 through a hydrogen bond. Residues at positions 4, 5, and 6 showed hydrophobic interactions with Leu32, Val36, Trp335, Tyr338, and Phe435. The N-terminal residues extended towards the active site lid and produced hydrophobic interactions with Ala65, Ala94, Leu98, Ala101, Tyr111, Leu115, Trp201, and Val495 (Fig. 3C and Supplementary Fig. 3B).

#### Insulin-regulated aminopeptidase

**Figure 4:**
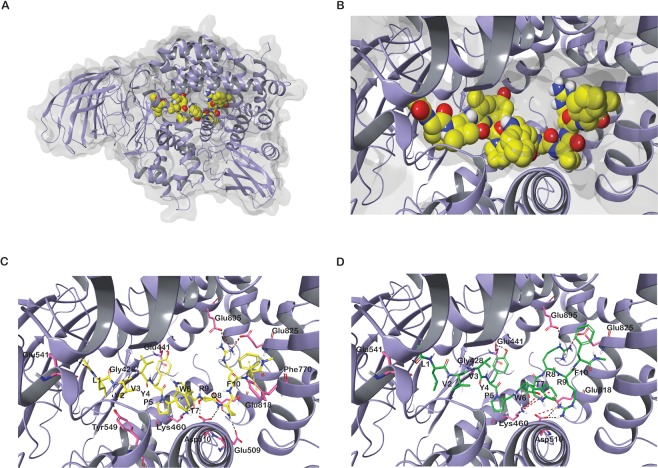
(**A**) Three dimensional structure of IRAP. (**B**) Binding pocket of IRAP. (**C**) LVVYPWTQRF (non-camel hemorphin) docked in the binding pocket of IRAP. (**D**) LVVYPWTRRF (camel hemorphin) docked in the binding pocket of IRAP. Hydrogen bonds are represented by black dotted lines and cation-π interactions are represented by red dotted lines.

The MM-GBSA binding free energy calculations performed for the different docking conformations of LVVYPWTQRF revealed that the pose with GScore −14.86 kcal/mol exhibited the highest MM-GBSA binding free energy of −148.04 kcal/mol. The N-terminal of both peptides bound in the S1 pocket, which is adjacent to the active site. Figure 4C shows the binding mode for non-camel LVV-hemorphin-7. In the predicted binding mode, the first three residues of LVV-hemorphin-7 occupied the S1 pocket and formed hydrogen bond with Leu541, Tyr549, and Gly428. All three residues formed hydrophobic interactions with residues in the S1 pocket - Tyr272, Pro296, Phe425, Ala427, Ala429, Met430, Phe544, and Tyr961. The aromatic residues Tyr4 and Trp6 formed hydrogen bonds with Glu441 and Lys460, respectively, and showed hydrophobic interactions with Ala453, Leu457, Ile461, Tyr495, Ala514, and Phe550. The C-terminal residues formed hydrophobic interactions with Ala763, Leu769, Phe770, Ala822, and Phe826. Thr7 formed a hydrogen bond with Asp510, Gln8 formed hydrogen bonds with Glu509, and Glu818 and Arg9 displayed hydrogen bonds with the side chain of residues Glu825 and Glu895 (Fig. 4C and Supplementary Fig. 4A). The binding score and interacting residues are summarized in Table 1.

The camel LVV-hemorphin-7 exhibited a similar binding mode with the N-terminal binding in the S1 pocket. The first three residues of the N-terminal bound in the S1 pocket and showed hydrophobic interactions with Tyr272, Pro296, Ala427, Ala429, Met430, Phe544, Tyr549, Pro957, and Tyr961. The amino group of Gly428 of the GAMEN loop (Gly428-Ala429-Met430-Glu431-Asn432) produced a hydrogen bond with Val2 residue in the N-terminal. The residues at positions 4, 5, and 6 formed hydrophobic interactions with the active site residues Leu457, Ile461, Tyr495, Ala514, and Phe550 and Trp6 formed a hydrogen bond with Lys460. Arg8 formed two hydrogen bonds with the side chain of Glu825 and Glu895, and Arg9 formed hydrogen bonds with Asp510 and Glu818. The C-terminal residues formed hydrophobic interactions with Leu769, Ala822, and Phe826 (Fig. 4D and Supplementary Fig. 4B). The best binding pose of camel LVV-hemorphin-7 with IRAP had a GScore of −13.65 kcal/mol and binding free energy of −163.03 kcal/mol (Table 1).

### Molecular dynamics simulations

To evaluate the stability and dynamics of the docked complexes, triplicate all-atom MD simulations were performed using Desmond^48^.

#### Simulations of camel and non-camel LVV-hemorphin-7 bound to MOR

**Figure 5:**
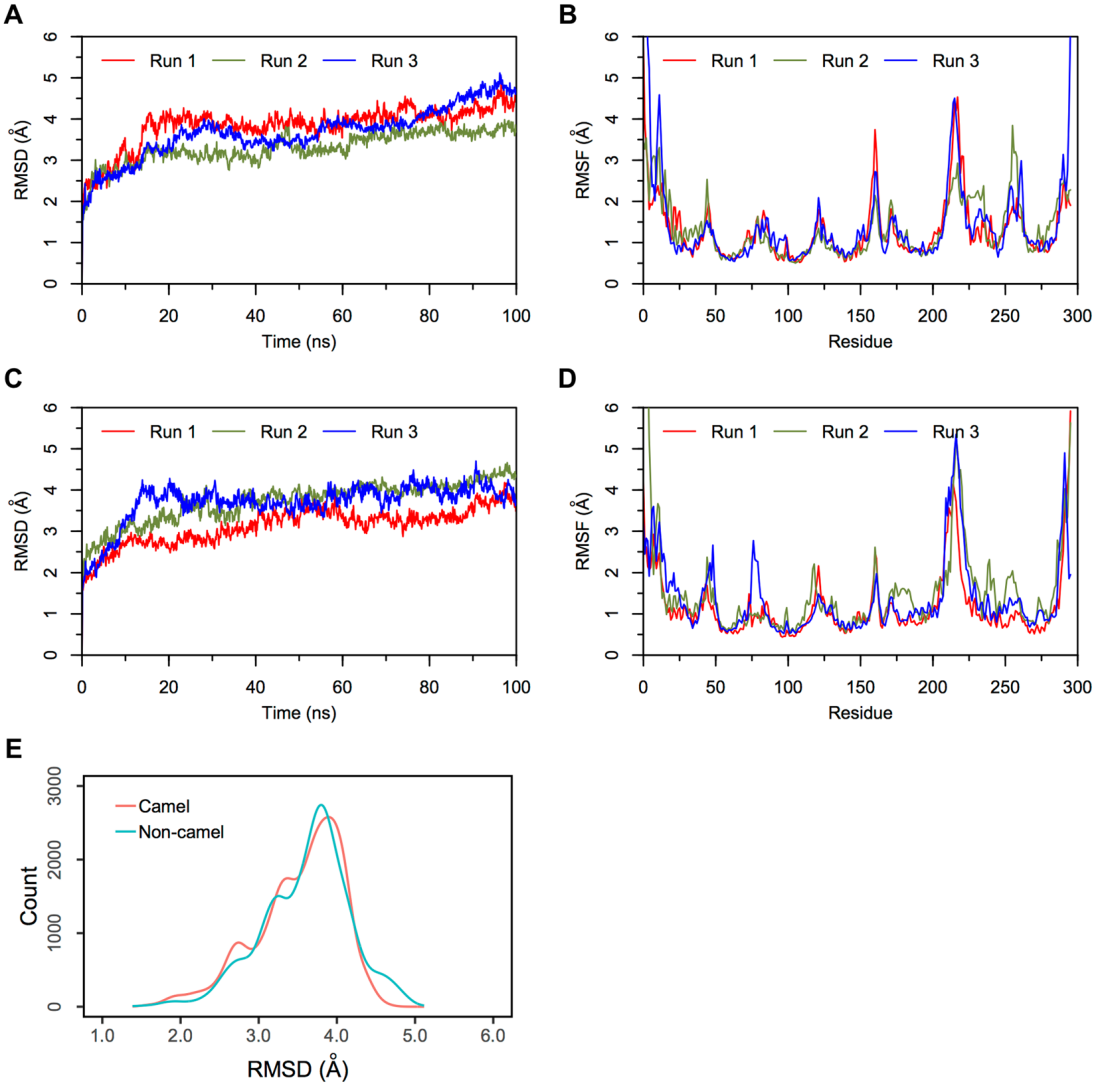
RMSD and RMSF plots of triplicate 100 ns simulations of MOR. Data from the three runs are plotted with red, blue and green lines. (**A**) RMSD of protein Cα atoms from the MOR-LVVYPWTQRF simulations. (**B**) RMSF of protein Cα atoms from the MOR-LVVYPWTQRF simulations. (**C**) RMSD of protein Cα atoms from the MOR-LVVYPWTRRF simulations. (**D**) RMSF of protein Cα atoms from the MOR-LVVYPWTRRF simulations. (**E**) Density functions corresponding to the distribution of RMSD values from triplicate hemorphin-bound simulations.

**Figure 6:**
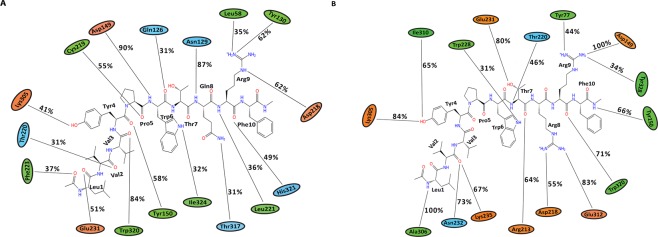
Average percentage of equilibrium simulation time during which MOR residues maintain contact with non-camel and camel LVV-hemorphin-7 from three 100 ns simulations. For equilibrium simulation data, the first 50 ns of run 1 was discarded, while the first 30 ns of runs 2 and 3 were discarded. Histograms representing the interaction from each of the 3 simulations can be found in Supplementary Fig. 5. Charged, hydrophobic and polar amino acids are represented with orange, green and blue color respectively. (**A**) Average percentage of time an MOR residue maintains contact with LVVYPWTQRF. (**B**) Average percentage of time an MOR residue maintains contact with LVVYPWTRRF.

Triplicate 100 ns equilibrium MD simulations of the non-camel LVV-hemorphin-7 bound to modelled MOR structure embedded in a dipalmitoylphosphatidylcholine (DPPC) membrane remained stable with a protein Cα root mean square deviation (RMSD) under 5 Å (Fig. 5A). The per-residue root mean square fluctuation (RMSF) showed that the third intracellular (IL-3) and extracellular loop (EL-2) regions were the most flexible while the membrane-embedded regions were found to be the least flexible (Fig. 5B). The peptide interacted with residues on TM3 and TM7 in MD simulations. Tyr4 and Trp6 maintained hydrogen bond and hydrophobic interactions with Tyr150 and Asp149 respectively on TM3 and Leu1 interacted with the TM5 residue Glu231. Tyr4 interacted with Lys305 while residue Val3, Trp6, and Arg9 maintained its interactions with Trp320, Ile324 and His321 respectively on TM7 during the simulations. The average percentage of equilibrated simulation time during which the non-camel LVV-hemorphin-7 interacted with MOR residues is given in Fig. 6A. Contributions from individual simulations are provided in Supplementary Fig. 5A.

In the camel LVV-hemorphin-7 bound simulations, the protein structure stabilized with RMSD under 4.5 Å in all three simulations (Fig. 5C). A distribution of the RMSD values obtained from the camel and non-camel hemorphin bound simulations is shown in Fig. 5E. EL-2 and IL-3 regions showed more fluctuations. More specifically, IL-3 showed more fluctuations compared to the MOR/non-camel LVV-hemorphin-7 complex (Fig. 5D). Camel LVV-hemorphin-7 showed interactions with residues from TM2, TM3, TM5, TM6, TM7 and the β-sheet. Arg9 interacted with Asp149 for the full duration of all three simulations while Phe10 interacted with Tyr150 during the simulations. LVVYPWTRRF also interacted with TM5 residues Glu231, Asn232, and Lys235. Leu1 and Tyr4 residues maintained continuous interactions with Ala306 and Lys305, respectively, on TM6 and the C-terminal residues interacted with Trp320, and Tyr328 on TM7. It also produced good interactions with Ile310 and Glu312 on the third extracellular loop. The average percentage of equilibrated simulation time during which the non-camel LVV-hemorphin-7 interacted with MOR residues is given in Fig. 6B. Contributions from individual simulations are provided in Supplementary Fig. 5B.

#### Simulations of camel and non-camel LVV-hemorphin-7 bound to ACE

**Figure 7:**
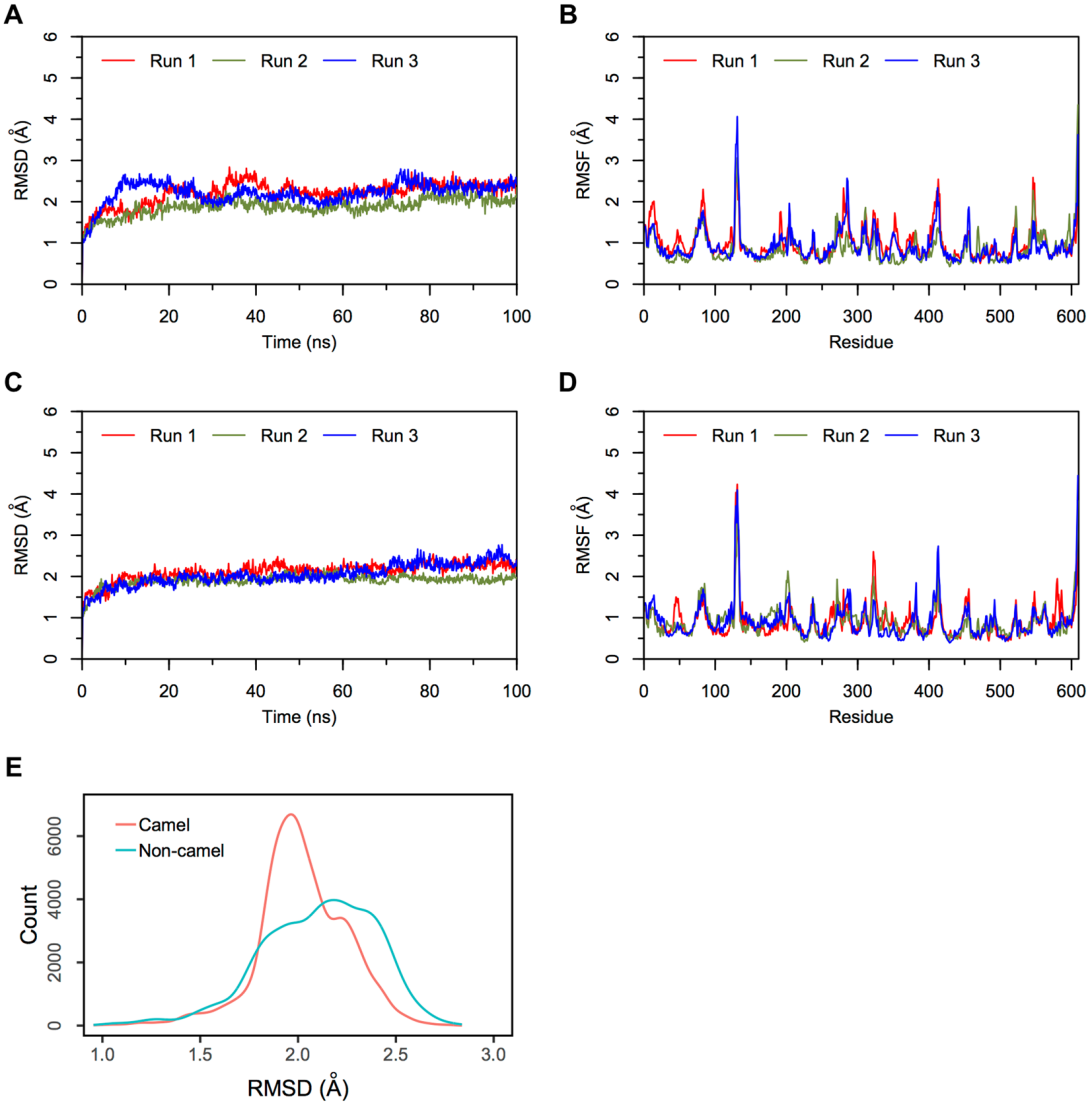
RMSD and RMSF plots of triplicate 100 ns simulations of ACE. Data from the three runs are plotted with red, blue and green lines. (**A**) RMSD of protein Cα atoms from the ACE-LVVYPWTQRF simulations. (**B**) RMSF of protein Cα atoms from the ACE-LVVYPWTQRF simulations. (**C**) RMSD of protein Cα atoms from the ACE-LVVYPWTRRF simulations. (**D**) RMSF of protein Cα atoms from the ACE-LVVYPWTRRF simulations. (**E**) Density functions corresponding to the distribution of RMSD values from triplicate hemorphin-bound simulations.

**Figure 8:**
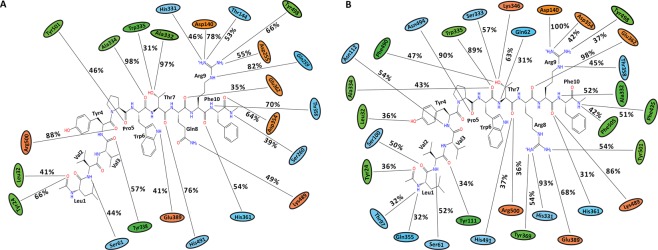
Average percentage of equilibrium simulation time during which ACE residues maintain contact with non-camel and camel LVV-hemorphin-7 from three 100 ns simulations. For equilibrium simulation data, the first 30 ns of each of the three simulations were discarded. Histograms representing the interaction from each of the 3 simulations can be found in Supplementary Fig. 6. Charged, hydrophobic and polar amino acids are represented with orange, green and blue color respectively. (**A**) Average percentage of time an ACE residue maintains contact with LVVYPWTQRF. (**B**) Average percentage of time an ACE residue maintains contact with LVVYPWTRRF.

Molecular dynamics simulations of ACE with camel and non-camel LVV-hemorphin-7 showed stable interactions throughout three 100 ns simulations. In the non-camel LVV-hemorphin-7 bound simulation, the ACE structure stabilized with RMSD under 3 Å (Fig. 7A). Most of the residues in the protein produced very limited fluctuation throughout the simulations (Fig. 7B). The C-terminal residues of hemorphin were stabilized in specific subsites of the active site by forming hydrogen bonds and hydrophobic interactions. C-terminal residues Gln8, Arg9, and Phe10 maintained the interaction with Asp140, Thr144, Asp255, Gln259, His331, His361, and Lys489. MD trajectory analysis showed that Arg9 occupied the S2 subsite by interacting with Asp255 and His331, as well as Gln259. The side chain of Arg9 interacted with Asp140 in the S1′ subsite. Trp6 and Thr7 interacted with residues Ala334, Trp335, Glu389, and His491, indicating its stable presence in S1. The non-camel LVV-hemorphin-7 also interacted with Tyr24, Ser61, Ser260, Tyr338, Thr358, Arg500, and Tyr501 during the simulations. The average percentage of equilibrated simulation time during which the non-camel LVV-hemorphin-7 interacted with ACE residues is given in Fig. 8A. Contributions from individual simulations are provided in Supplementary Fig. 6A.

In the camel LVV-hemorphin-7 bound simulations, the protein structure showed structural stability throughout the simulations and the RMSD plot indicated that the structures stabilized under 2.5 Å after the initial few nanoseconds of the simulation (Fig. 7C). Residues of the protein showed lower fluctuations (Fig. 7D). The secondary structure composition remained intact throughout the course of the simulations. A distribution of the RMSD values obtained from the camel and non-camel hemorphin bound simulations are shown in Fig. 7E. Camel LVV-hemorphin-7 remained stably bound by maintaining hydrogen bonds between the arginine residues (Arg8 and Arg9) located near the C-terminal and Asp140, His331, and Tyr369. The side chain of Arg9 interacted with Asp140 in the S1′ pocket for the full duration of all three simulations. The substituted Arg8 maintained the hydrogen bond with His331, Tyr369, and Glu389, and also maintained the hydrophobic interaction with Tyr501 in the S1 pocket. Additionally, the peptide also interacted with the HEXXH motif, specifically His361 and Glu362, during the simulations. The average percentage of equilibrated simulation time during which the non-camel LVV-hemorphin-7 interacted with ACE residues is given in Fig. 8B. Contributions from individual simulations are provided in Supplementary Fig. 6B.

#### Simulations of camel and non-camel LVV-hemorphin-7 bound to IRAP

**Figure 9:**
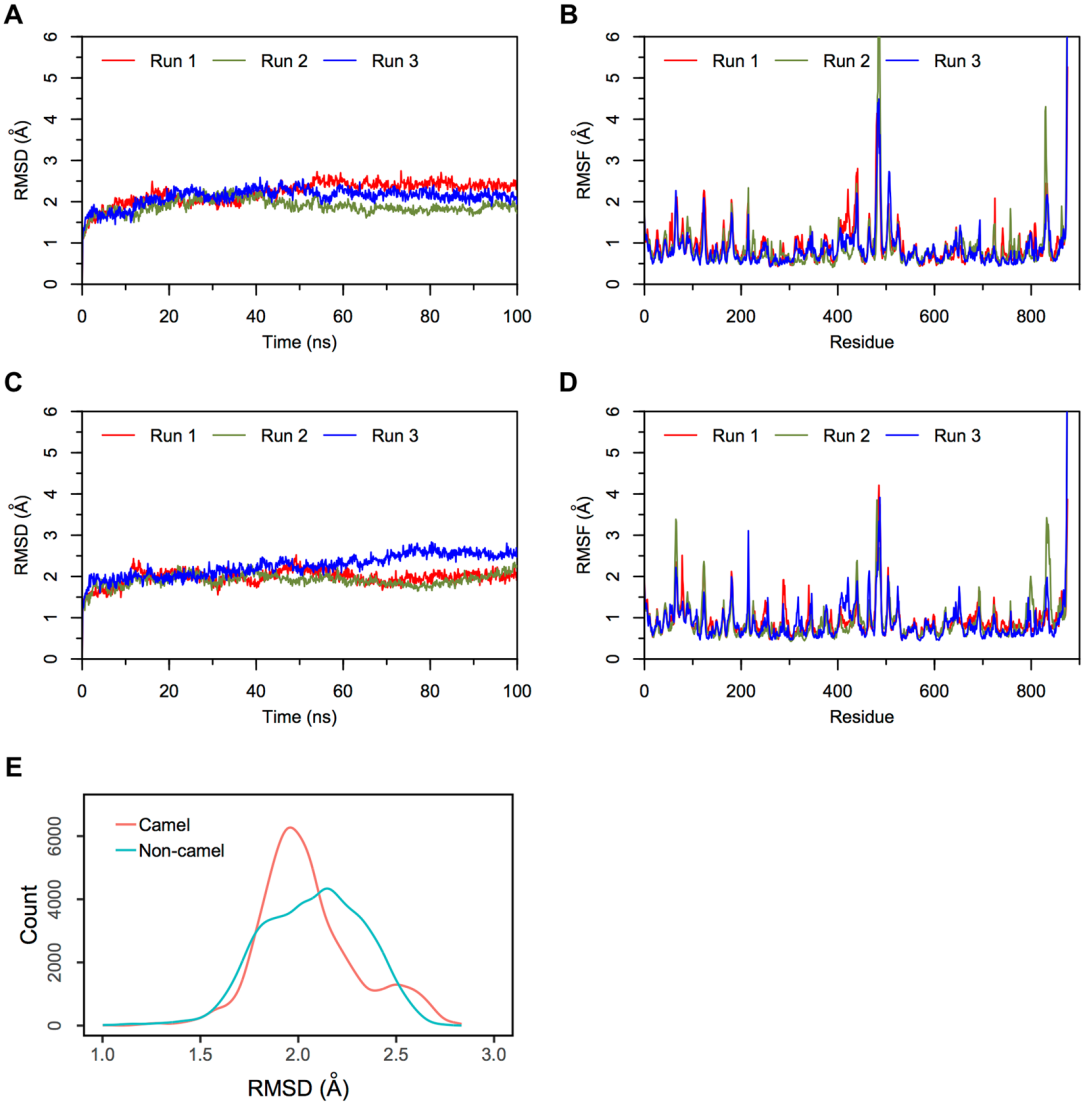
RMSD and RMSF plots of triplicate 100 ns simulations of IRAP. Data from the three runs are plotted with red, blue and green lines. (**A**) RMSD of protein Cα atoms from the IRAP-LVVYPWTQRF simulations. (**B**) RMSF of protein Cα atoms from the IRAP-LVVYPWTQRF simulations. (**C)** RMSD of protein Cα atoms from the IRAP-LVVYPWTRRF simulations. (**D**) RMSF of protein Cα atoms from the IRAP-LVVYPWTRRF simulations. (**E**) Density functions corresponding to the distribution of RMSD values from triplicate hemorphin-bound simulations.

**Figure 10:**
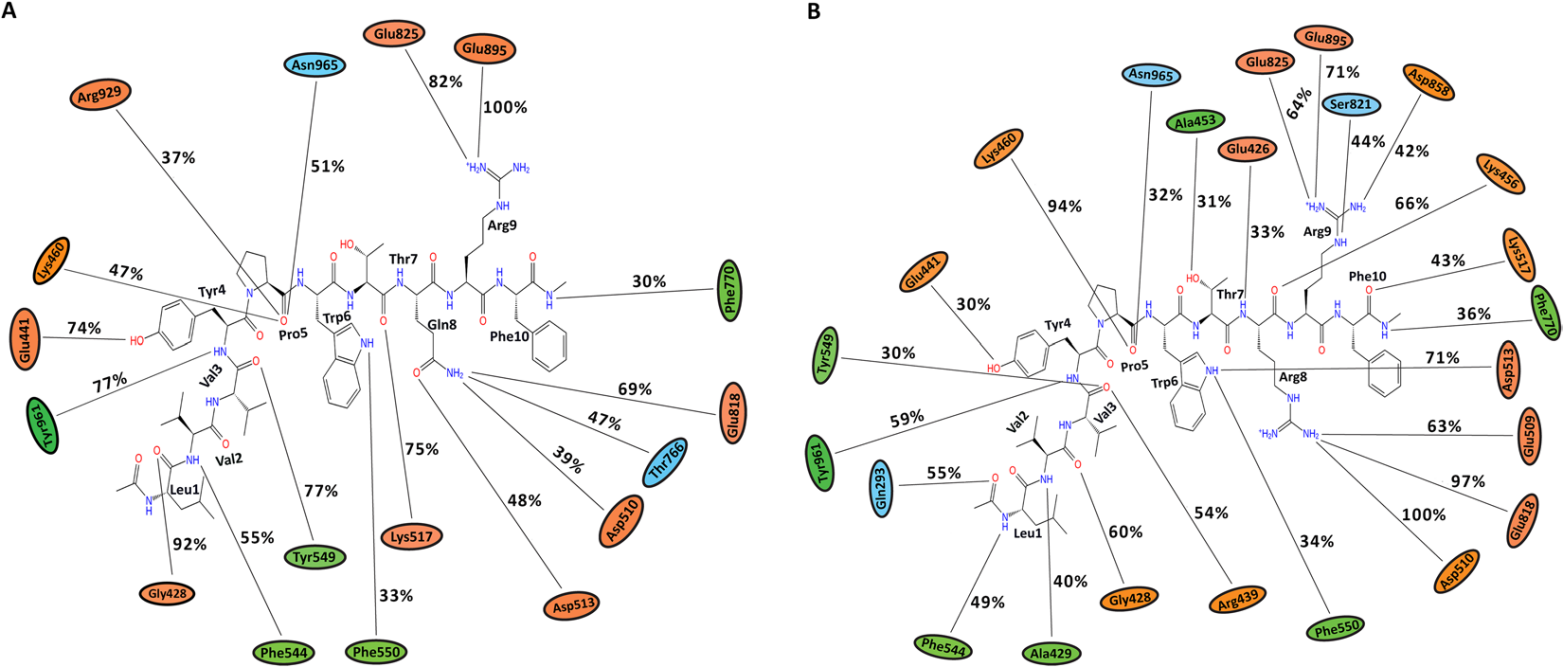
Average percentage of equilibrium simulation time during which IRAP residues maintain contact with non-camel and camel LVV-hemorphin-7 from three 100 ns simulations. For equilibrium simulation data, the first 30 ns of each of the three simulations were discarded. Histograms representing the interaction from each of the 3 simulations can be found in Supplementary Fig. 7. Charged, hydrophobic and polar amino acids are represented with orange, green and blue color respectively. (**A**) Average percentage of time an IRAP residue maintains contact with LVVYPWTQRF. (**B**) Average percentage of time an IRAP residue maintains contact with LVVYPWTRRF.

The stability of the bound peptide was studied using 100 ns molecular dynamics simulation runs. In non-camel LVV-hemorphin-7 bound simulations, the IRAP structures stabilized with RMSD under 2.5 Å (Fig. 9A). There were limited fluctuations in the protein residues and the secondary structure remained intact throughout these simulations (Fig. 9B). The N-terminal residues of non-camel LVV-hemorphin-7 were stabilized in the S1 pocket with hydrogen bonds as well as hydrophobic interactions. Leu1, Val2 and Val3 maintained hydrogen bonds with Tyr549 and Gly428 during the simulations and also interacted with Phe544. Tyr4, Pro5, and Trp6 maintained their interactions with Glu441, Phe550, and Asn965. While C-terminal residues maintained their interactions with the residues Asp510, Asp513, Glu818, Gu825, and Glu895. During the simulations, new contacts were formed between the first three residues of N-terminal and Trp6 with Arg929, and Asn965. The average percentage of equilibrated simulation time during which the non-camel LVV-hemorphin-7 interacted with IRAP residues is given in Fig. 10A. Contributions from individual simulations are provided in Supplementary Fig. 7A.

Camel LVV-hemorphin-7 simulations remained stable with an RMSD under 2.75 Å (Fig. 9C). There were limited fluctuations in IRAP residues throughout the camel peptide-bound simulations (Fig. 9D). The secondary structure elements also remained intact throughout the simulations. A distribution of the RMSD values obtained from the camel and non-camel hemorphin bound simulations are shown in Fig. 9E. The N-terminal of camel LVV-hemophin-7 was embedded in the S1 pocket of IRAP by maintaining hydrogen bonds and hydrophobic interactions. Val2 maintained a hydrogen bond with Gly428 in the GAMEN loop during these simulations and the N-terminal residues maintained the hydrophobic interactions with Ala429, Phe544, Tyr549, and Tyr961. Pro5 produced sustained interactions with Lys460, while Trp6 interacted with Asp513. The substituted Arg8 showed strong hydrogen bonds with the negatively charged residues Glu509, Asp510 and Glu818. Arg9 and Phe10 also interacted with Lys517, Phe770, Glu825 and Glu895 stabilizing it in the docked pose. The average percentage of equilibrated simulation time during which the non-camel LVV-hemorphin-7 interacted with IRAP residues is given in Fig. 10B. Contributions from individual simulations are provided in Supplementary Fig. 7B.

## Discussion

Several *in vivo* and *in vitro* studies have reported the therapeutic potential of hemorphin^16,17,26,32^. The ability of LVV-hemorphin-7 to bind to several proteins with high affinity emphasizes the importance of understanding the binding mode and activity of this peptide at the molecular level. Computational modeling often complements experimental studies. This study employed computational approaches to investigate the binding mode of both camel and non-camel LVV-hemorphin-7 with MOR, ACE, and IRAP to understand how the peptide interacts with these unrelated proteins.

Camel and non-camel LVV-hemorphin-7 adopted different poses in the MOR. The key residues involved in the binding are consistent with previous experimental and theoretical studies^49–51^. Both camel and non-camel LVV-hemorphin-7 were found to interact with the acidic residue Asp149 of MOR with a hydrogen bond (Fig. 6A,B). Camel LVV-hemorphin-7 interacted for the entire duration of the three simulations while the interaction was sustained for an average of 90% of the three non-camel LVV-hemorphin-7 simulations. This finding supports various structural models and site-directed mutagenesis studies that highlighted the important role played by Asp149 (Asp147 in murine MOR) in opioid ligand recognition and the interaction with various opioid ligands^49,52–55^. Therefore, consideration should be given to this vital interaction for future novel design of MOR ligands. Hydrophobic interactions were formed between camel LVV-hemorphin-7 and Tyr328, which is consistent with a previous report that indicated that hemorphin interacts with Tyr326 of mouse MOR^49,51,56^. The interaction with Trp320 on TM7 is also consistent with previous studies^51,57,58^. Interestingly, camel LVV-hemorphin-7 interacted with key residues in the binding pocket for a longer duration than non-camel hemorphin (Fig. 6). LVVYPWTRRF interacted for a longer duration than LVVYPWTQRF with Thr220, Glu231, and Tyr328 in the human MOR model. The corresponding residues in the murine MOR – Thr200, Glu299, and Tyr326 – have been shown to interact with agonists^49,51,56,58^. RMSF values were found to be high for IL-3 residues when bound to camel LVV-hemorphin-7. Major interactions for camel LVV-hemorphin-7 on MOR were observed in TM3, TM6, and TM7 which has been suggested to contain the ionic lock responsible for GPCR activation^59^. As expected, the peptide-bound simulations led to greater flexibility of the IL-3 region, which is in agreement with downstream protein complex interactions and signaling pathways activated by receptor agonists^60^. Camel LVV-hemorphin-7 bound simulations had a lower RMSD when compared to non-camel LVV-hemorphin-7 (Fig. 5E). EL-2 showed lower fluctuations upon binding to both camel and non-camel-LVV-hemorphin-7. This may help with binding and peptide selectivity. This is consistent with various experimental studies of mutagenesis which confirmed the role of EL-2 in ligand allosteric and orthosteric activation^61–63^. Moreover, residues Trp320 and Tyr328 are important in controlling biased MOR signaling and are critical in effector coupling^64^. Interestingly, camel LVV-hemorphin-7 interacted with both residues, while the non-camel LVV-hemorphn-7 interacted with only Trp320. Thus, it is possible that camel hemorphin could produce their effect on MOR as a result of ligand bias.

Molecular modeling and simulation were used to elucidate the behavior of the binding of LVV-hemorphin-7 to ACE. Both camel and non-camel peptides were found to stably bind in the active site of ACE with the C-terminal bound in specific subsites of the active site and the N-terminal oriented towards the active site lid. Camel LVV-hemorphin-7 bound simulations had a lower RMSD when compared to non-camel LVV-hemorphin-7 (Fig. 7E). The ACE active site has three subsites – S1, S2, and S1′. S1 includes Ala332, Glu362, and Tyr501; S2 includes Gln259, His331, Lys489, His491, and Tyr498, while S1′ contains the residue Glu140 (which is mutated to Asp140 in PDB ID: 2XYD)^65^. In both ACE non-camel and camel LVV-hemorphin-7 complexes, the C-terminal residues from positions 7 to 10 occupied the S1, S2, and S1′ subsites (Fig. 3). Furthermore, ACE has a Zn^2+^ ion in its active site that coordinates with His361, His365, and Glu389. These regions are essential for the formation of ACE–inhibitor complex^66^. In the docked poses of non-camel LVV-hemorphin-7, the C-terminal Arg9 and Phe10 anchored the peptide in the active site through a hydrogen bond interaction with His331 in the S2 subsite. Thr7 and Gln8 bound in the S1 subsite and maintained the polar interaction with His361 and His491 (Fig. 8A). The positioning of camel hemorphin in the ACE active site was different from non-camel hemorphin. Specifically, the C-terminal was positioned differently in the S1′ and S2 pockets (Fig. 3). Arg9 and Phe10 occupied the S2 subsite with the side chain of Phe10 stabilized by interactions with S1 and S2 subsite residues Ala332, His361, and Lys489. The side chain of Arg9 formed a hydrogen bond with Asp140 present in the S1′ subsite for the full duration of all three simulations (Fig. 8B). Additionally, Arg8 showed more interactions with active site residues than Gln8 in non-camel LVV-hemorphin-7 (Figs 3 and 8). Arg8 interacted with His331 in the S2 subsite and with Tyr501 in the S1 subsite, as well as Glu362 and Glu389 potentially displaying the ability to replace the active site Zn^2+^ ion. Additionally, camel LVV-hemorphin-7 showed more sustained interactions than non-camel LVV-hemorphin-7 with His331, Glu362, Tyr369, and Lys489 throughout the simulation runs which are important for ACE-inhibitor complex formation. This could be the reason for the better binding free energy in the docked pose of camel hemorphin in the ACE active site. Importantly, known inhibitors of ACE interacts with similar residues – His331, Ala332, Glu362, and Tyr501– indicating that these amino acids play a major role in binding to ACE^67–69^. Here, camel LVV-hemorphin-7 interacted for a longer duration to these residues than non-camel LVV-hemorphin-7, which could support its role as a better ACE inhibitor. Hydrophobic interactions between the peptide and key amino acids in ACE also contributed to the stabilization of the binding pose.

Camel LVV-hemorphin-7 produced similar binding interactions with IRAP when compared to non-camel LVV-hemorphin-7. In the predicted binding pose of non-camel and camel LVV-hemorphin-7, the N-terminal residues were stabilized in the S1 pocket by maintaining hydrogen bonds with Gly428 and Tyr549 (Fig. 10). Tyr549 is highly conserved in the M1 family of metalloproteases and was shown to be important for binding and stabilization of the catalytic transition state^70^. Furthermore, it also interacted with Phe544 and Tyr961 in the active site. The interaction with Phe544 was previously reported as a key interaction for substrate and ligand binding^71,72^. IRAP is characterized by a catalytic domain that contains two conserved motifs, the HEXXH Zn^2+^ binding motif and the GXMEN exopeptidase motif. Mutational analysis of these motifs indicated that Gly428, Ala429, and Asn432 are important for binding of both peptide substrates and inhibitors, and also confirmed that peptide IRAP inhibitors competitively bind to its catalytic site^73^. In this study, the N-terminal residues of camel LVV-hemorphin-7 interacted with the GAMEN loop residues Gly428 and Ala429, while non-camel LVV-hemorphin-7 interacted with only Gly428 (Fig. 10). Specifically, Val2 of camel LVV-hemorphin-7 interacted with Gly428 and Ala429 (Fig. 10B). Val3 of both non-camel and camel LVV-hemorphin-7 interacted with Tyr549. Val3 is crucial for LVV-hemorphin-7 binding to IRAP; removal of Val3 leads to the abolition of binding to IRAP^74^. Tyr4 and Trp6 interacted with Glu441 and Phe550. It was reported that monosubstitutions of Tyr4 and Trp6 with alanine resulted in a 10-fold reduction in affinity^74^. N-terminal residues of hemorphin are crucial for IRAP inhibition forming strong interactions with critical IRAP residues. It was shown that the C-terminal deletion of LVV-hemorphin-7 (Thr7, Gln8, Arg9, and Phe10) did not significantly affect their affinity for IRAP. In fact, a modest decrease in affinity was reported with deletion of Arg9 residue in cerebellar membranes^74^. In the simulations, LVVYPWTQRRF residues 8 and 9 were observed to form sustained interactions with charged Lys456, Glu509, Asp510, Asp513, and Glu818 when compared to LVVYPWTQRF bound simulations (Fig. 10A,B). These interactions could result in a better affinity of camel LVV-hemorphin-7 in the IRAP catalytic pocket.

## Materials and Methods

### Multiple sequence alignment of hemoglobin beta protein

Hemoglobin beta protein sequences of *Homo sapiens* (NP_000509), *Camelus dromedarius* (XP_010988890), *Equus caballus* (XP_001504239), *Bos taurus* (NP_776342), *Sus scrofa* (NP_001138313), *Ovis aries* (NP_001091117), *Pan troglodytes* (NC_036890) and *Oryctolagus cuniculus* (NP_001075729) were retrieved from NCBI protein database. Multiple sequence alignment of these sequences was performed using ClustalW^75^.

### Protein structure identification and preparation

To dock the peptides to the human MOR, a homology model was generated using Schrödinger Prime^76^. A Protein Data Bank (PDB) BLAST search was performed to identify homologous protein structures. The three-dimensional homology model was generated for the active human MOR (UniProt accession: P35372) using the X-ray crystallographic structure of the active MOR from *Mus musculus* (PDB ID: 5C1M) as the template. Loops were refined and the structure was verified using the protein refinement module of Schrödinger Prime. The three-dimensional structure of ACE (PDB ID: 2XYD) and IRAP (PDB ID: 5MJ6) were downloaded from the PDB^77^. The downloaded structures were prepared using the Protein Preparation Wizard of Schrödinger Suite^78^. Protein preparation was performed to remove unwanted water molecules, metals, and cofactors. The procedure simplified multimeric complexes, created disulfide bonds, assigned bond orders properly, adjusted ionization states, and fixed the orientation of misoriented groups. Hydrogen atoms were added to the protein structures, and standard protonation states at pH 7 were used. The preprocessed structures were then optimized and minimized to generate geometrically stable structures^79^.

### Active site identification and grid generation

Receptor grids were generated for the prepared protein structures to assist with the docking. Receptor grids were generated with the default parameters for van der Waals scaling factor (1.00) and charge cutoff (0.25) employing the OPLS 2001 force field. A cubic search box was defined centered on the centroid of the binding site residues for each receptor.

### Glide standard precision docking

Schrödinger Glide’s Standard Precision (SP) method was used to dock peptides with default parameters. SP flexible ligand docking was carried out using Schrödinger Glide version 2016-4 with penalties applied for cis amide bonds^80^. Peptides were docked starting from multiple random conformations, which were generated by the ConfGen algorithm^81^. The poses were partially optimized using the standard OPLS 2005 molecular mechanics force field (SP minimization). Ten representative peptide conformations were selected after clustering the conformers generated. Finally, all ten poses were subjected to post-docking minimization in the gridded protein field. Top poses were rescored and ranked by the GlideScore (GScore) scoring function^82^. The best-docked pose with lowest GScore value was recorded for each peptide.

### Binding free energy calculation

The SP docking poses were subjected to molecular mechanics generalized Born surface area (MM-GBSA) to evaluate the binding free energy in an implicit solvent model. MM-GBSA binding energy was calculated using Schrödinger﻿ Prime employing the OPLS 2005 force field and the VSGB 2.0 implicit solvent model^76,83^. For the MM-GBSA calculations, the receptor was treated rigidly and the peptide was minimized.

### Molecular dynamics simulation

In order to confirm the stability of the binding pose of the non-camel and camel LVV-hemorphin-7 in the active site of the three proteins, triplicate MD simulations were performed using Desmond employing the OPLS 2005 force field^82^.

### Simulations of MOR

Three systems – peptide-free MOR, MOR with LVVYPWTQRF (non-camel hemorphin), and MOR with LVVYPWTRRF (camel hemorphin) – were prepared. Three runs each of the peptide bound simulations were performed with different initial velocities. The modeled structure of MOR and the complexes of MOR with non-camel and camel LVV-hemorphin-7 were embedded into a pre-equilibrated DPPC membrane in an orthorhombic box^84^. All systems were solvated with a water box, using SPC water model^85^, with a buffer distance of 10 Å. Counterions were added and sufficient number of ions were added to maintain a salt concentration of 0.15 M NaCl. The systems were subjected to steepest descent minimization with Desmond’s default protocol prior to performing MD simulations.

All three systems were first relaxed using the default relaxation protocol for membrane proteins^86^. The relaxation protocol consists of eight stages that included minimization with restraints on solute heavy atoms, minimization without any restraints, simulation with heating from 0 K to 300 K, H_2_O barrier and gradual restraining, simulation under NPT equilibration with H_2_O barrier with heavy atoms restrained, NPT equilibration of solvent and lipids, simulation under the NPT ensemble with protein heavy atoms restraint reduced from 10.0 to 2.0 kcal/mol, NPT equilibration with Cα atoms restrained at 2 kcal/mol, and simulation for 1.5 ns under the NPT ensemble with no restraints. After relaxation, unrestrained simulation run was performed for 100 ns for each system.

The simulations were performed under NPT ensemble using the Nose-Hoover thermostat to maintain a constant temperature of 300 K and isotropic Martyna-Tobias-Klein barostat to maintain the pressure at 1 atm^87,88^. The short-range Coulombic interactions were analyzed with a cut-off value of 9.0 Å using the short-range method. A time-reversible reference system propagator algorithm (RESPA) integrator was used with a time step of 2.0 fs^89^. The trajectories were saved at 100 ps intervals for analysis. After simulations were performed, RMSD, RMSF, and protein-ligand contacts were evaluated.

### Simulation of ACE and IRAP

The systems were solvated with SPC solvent model in an orthorhombic box to minimize the volume. Simulation systems were neutralized by adding sufficient number of Na^+^/Cl^−^ counterion and a salt concentration of 0.15 M NaCl was maintained. The systems were then minimized with Desmond’s default protocol before performing MD simulations. Three 100 ns simulations were performed for each system with different initial velocities. The simulations were performed under NPT ensemble using the Nose-Hoover thermostat to maintain a constant temperature of 300 K and isotropic Martyna-Tobias-Klein barostat to maintain the pressure at 1 atm^87,88^. The short-range coulombic interactions were analyzed with a cut-off value of 9.0 Å using the short-range method. After simulations were performed RMSD, RMSF, and protein-ligand contacts were evaluated.

## Conclusion

This study provides the first structural insight into the binding of LVV-hemorphin 7 with MOR, ACE and IRAP. Interestingly, among mammals, camel LVV-hemorphin-7 is unique due to an amino acid substitution. Results showed that camel LVV-hemorphin-7 produced more sustained interactions with all three proteins – MOR, ACE and IRAP – than non-camel LVV-hemorphin-7. Substitution of glutamine with an arginine in camel hemorphin seems to produce a more stable interaction in the proteins studied. Further studies could investigate the effect of camel LVV-hemorphin-7 at the cellular and molecular level for its potential as a therapeutic agent for memory loss, hypertension, and analgesia. Finally, it would also be interesting to extend the study to other proteins involved in the regulation of RAS homeostasis.

## Supplementary information

Supplementary Materials
