# Supplementary material for: Molecular insights into the interaction of hemorphin and its targets
Source: Sci Rep. 2019 Oct 14;9:14747. doi: 10.1038/s41598-019-50619-w (PMC6791854; doi:10.1038/s41598-019-50619-w)
Supplement: Supplementary file 1 — Supplementary Materials [file 41598_2019_50619_MOESM1_ESM.pdf]

Molecular insights into the interaction of hemorphin and its targets

Amanat Ali[a,†], Bincy Baby[a,†], Soja Saghar Soman[b] and Ranjit Vijayan[a,*]

[a]Department of Biology, College of Science, United Arab Emirates University, PO Box 15551, Al Ain, Abu Dhabi, United Arab Emirates.

[b]New York University Abu Dhabi, PO Box 129188, Abu Dhabi, United Arab Emirates

*Correspondence: Tel: +971 3 713 6302, Email: ranjit.v@uaeu.ac.ae

[†] These authors contributed equally.

Supplementary Materials

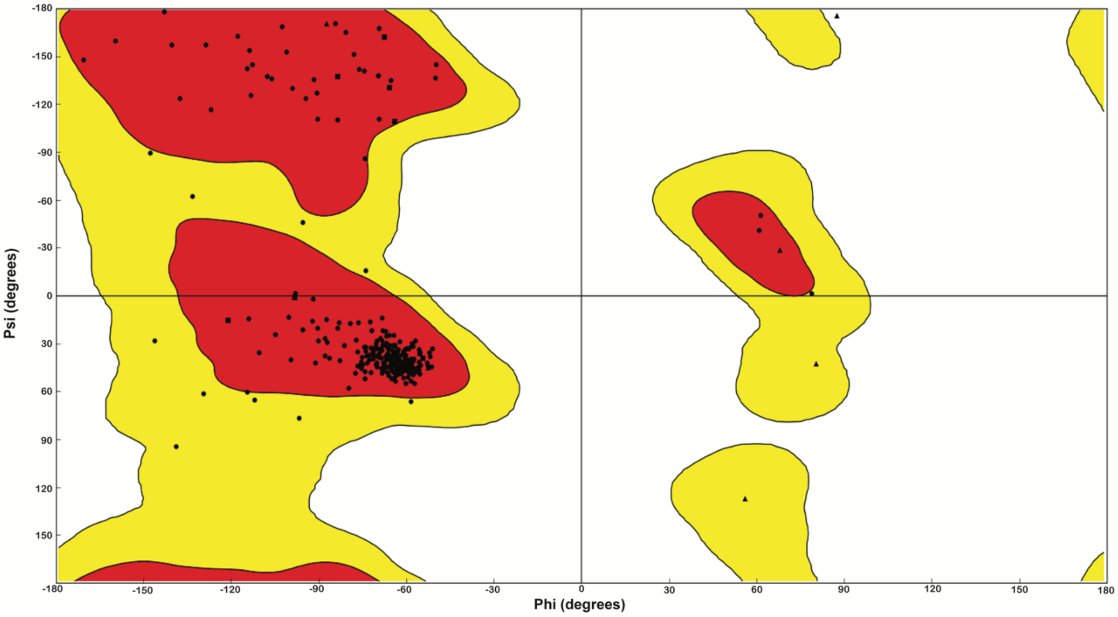

Supplementary Figure 1. Ramachandran plot of human MOR model.

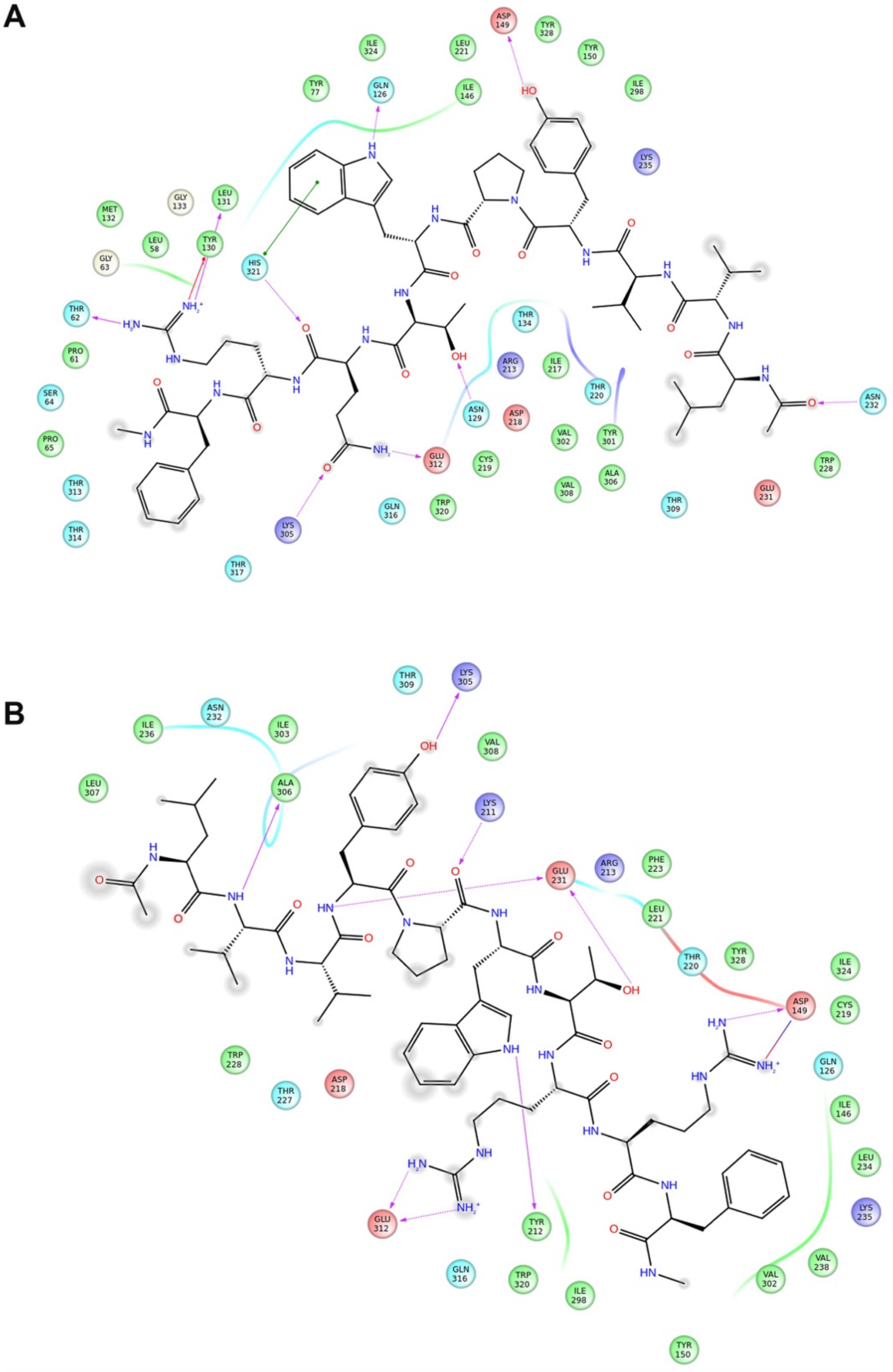

Supplementary Figure 2. Interaction of LVV-hemorphin-7 residues with MOR. A) MOR-LVVYPWTQRF. B) MOR-LVVYPWTRRF.

A

B

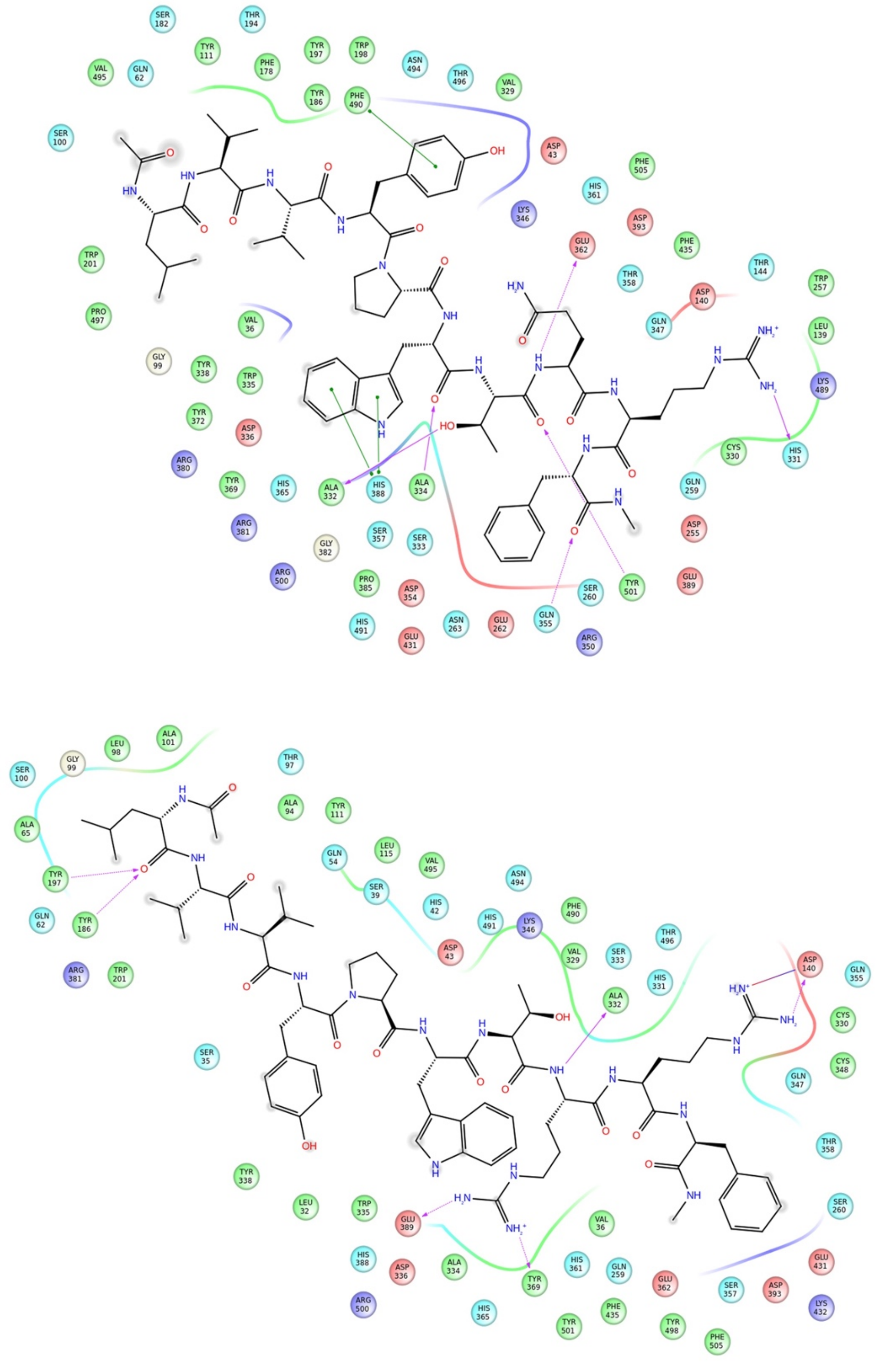

Supplementary Figure 3. Interaction of LVV-hemorphin-7 residues with ACE (PDB ID: 2XYD) A) ACE-LVVYPWTQRF. B) ACE-LVVYPWTRRF.

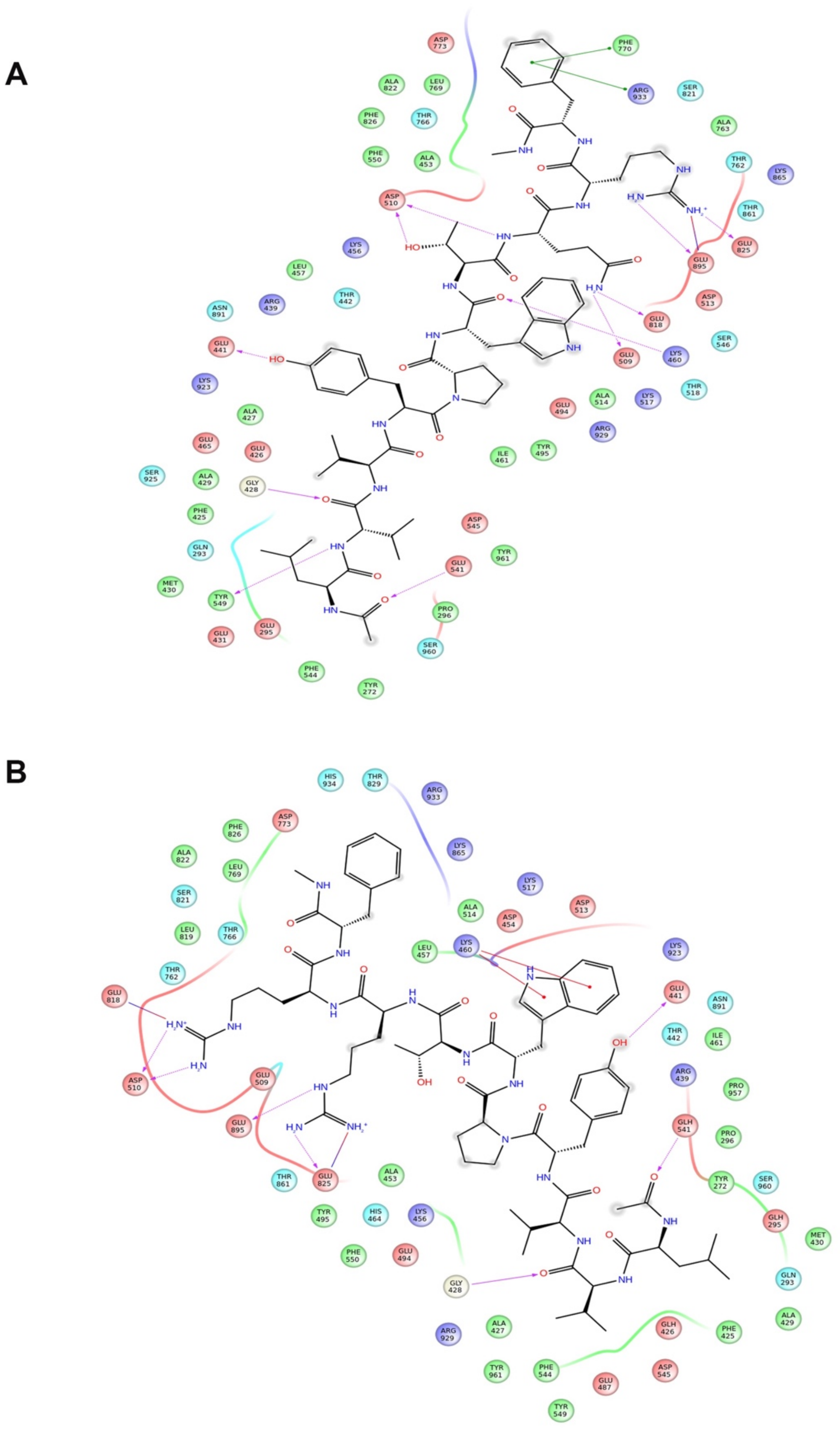

Supplementary Figure 4. Interaction of LVV-hemorphin-7 residues with IRAP (PDB ID: 5MJ6) A) IRAP-LVVYPWTQRF. B) IRAP-LVVYPWTRRF.

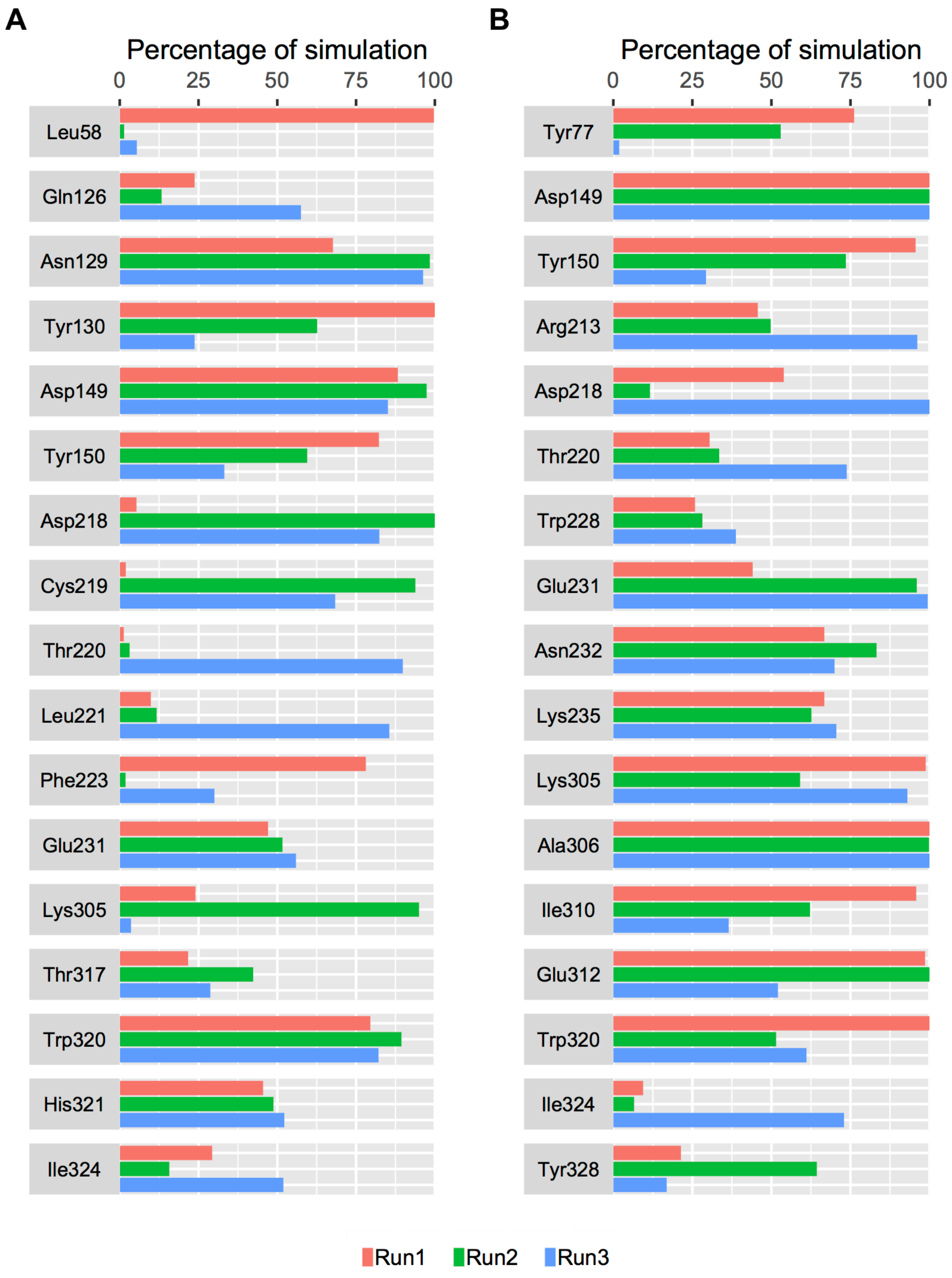

Supplementary Figure 5. Histograms of the percentage of simulation time where a MOR residue maintains contact with the hemorphin peptide in triplicate simulations. For equilibrium simulation data, the first 50 ns of run 1 was discarded, while the first 30 ns of runs 2 and 3 were discarded. A) MOR-LVVYPWTQRF. B) MOR-LVVYPWTRRF.

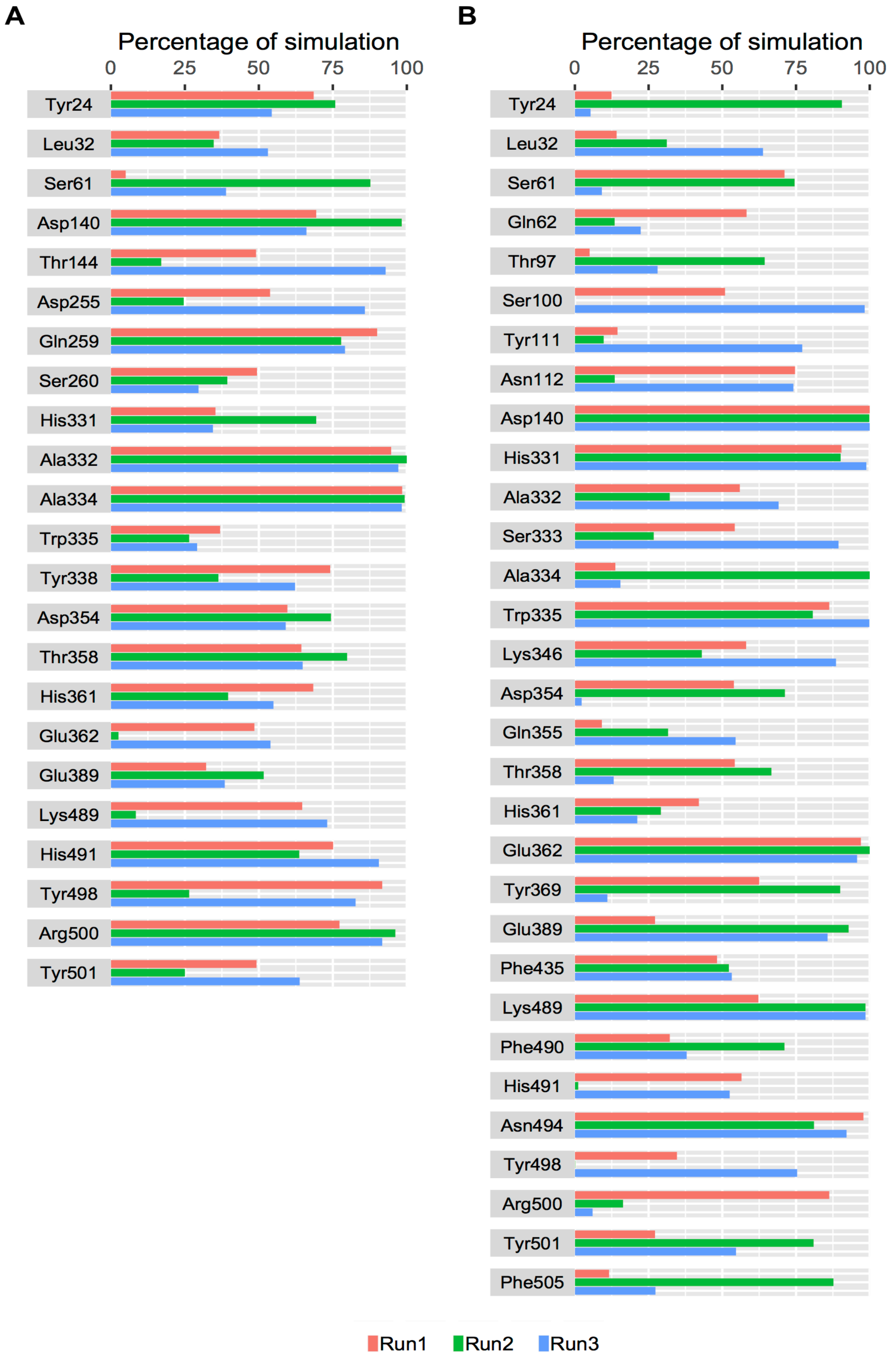

Supplementary Figure 6. Histograms of the percentage of simulation time where a ACE residue maintains contact with the hemorphin peptide in triplicate simulations. For equilibrium simulation data, the first 30 ns of each run was discarded. A) ACE-LVVYPWTQRF. B) ACE-LVVYPWTRRF.

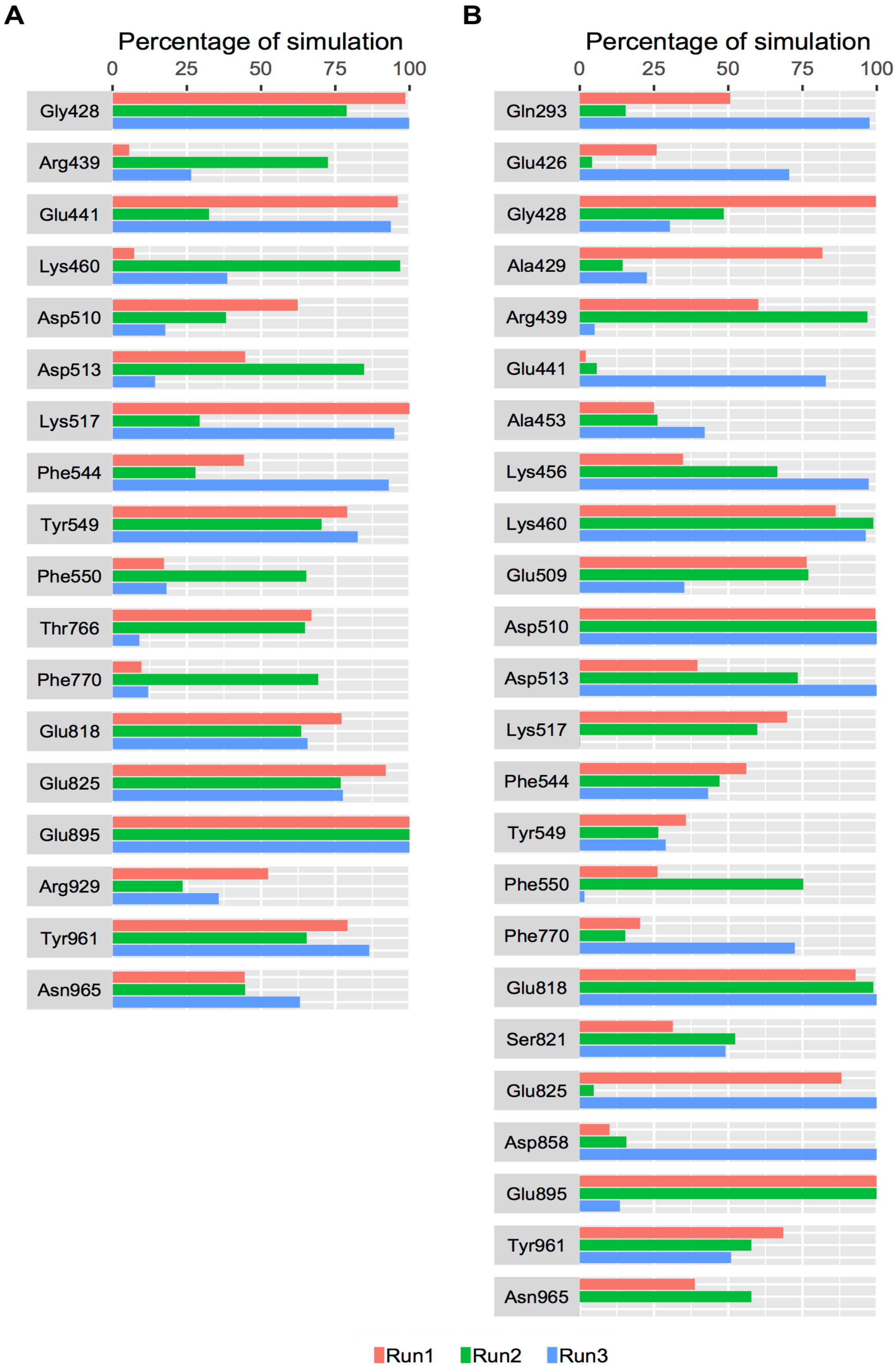

Supplementary Figure 7. Histograms of the percentage of simulation time where an IRAP residue maintains contact with the hemorphin peptide in triplicate simulations. For equilibrium simulation data, the first 30 ns of each run was discarded. A) IRAP-LVVYPWTQRF. B) IRAP-LVVYPWTRRF.
